# What Is Your Diagnosis? Superficial Cervical Lymph Node Cytology

**DOI:** 10.1111/vcp.70101

**Published:** 2026-04-15

**Authors:** Vinicius N. Hirata, Rúbia S. da Silva, Francisca B. Lang, Augusto de Oliveira, Cinthia M. de Andrade

**Affiliations:** ^1^ Department of Small Animal Clinic, Veterinary Teaching Hospital Federal University of Santa Maria Santa Maria Rio Grande do Sul Brazil

**Keywords:** cytopathology, lymph node, metastasis

## 1 Case Presentation

An 8‐year‐old spayed female Maltese dog presented to the Veterinary Hospital of the Federal University of Santa Maria (UFSM) due to a 1‐week history of diarrhea with mucus and an episode of vomiting on the day of the consultation. On physical examination, an enlargement was noted in the region of the right mandibular (2.5 × 2 × 1 cm) and superficial cervical (2.7 × 1.5 × 1.5 cm) lymph nodes, with an unknown duration of evolution. No other clinical findings were observed. Hemogram and serum biochemistry tests were requested, and no clinically significant abnormalities were found.

Fine‐needle biopsy (FNB) of both enlarged regions was performed. Slides were stained with aqueous Romanowsky stain, and cytologic analysis was performed. Cytology from the right superficial cervical region is exhibited in Figure 1.

**FIGURE 1:**
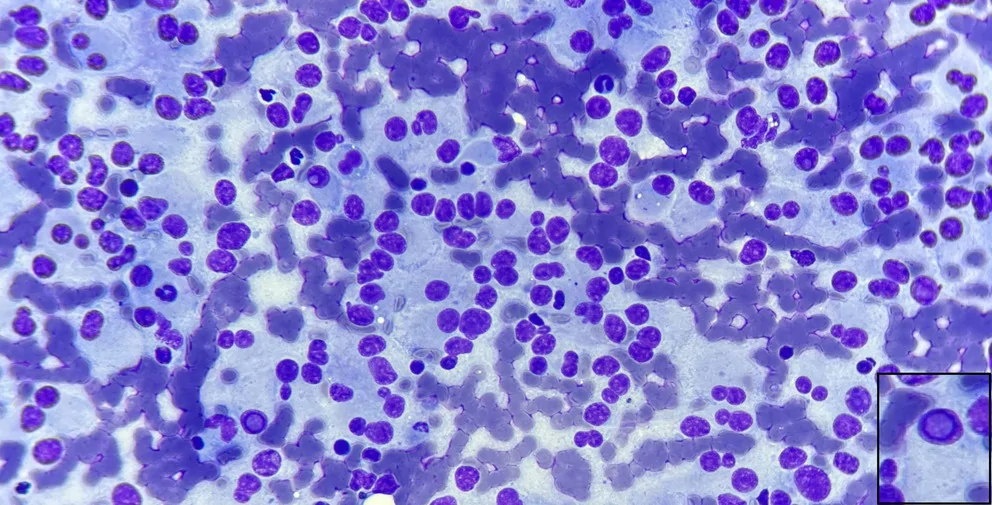
Photomicrograph of a fine‐needle biopsy from the right superficial cervical region of an 8‐year‐old spayed female Maltese dog. Aqueous Romanowsky stain, ×40 objective. Inset highlights an intranuclear pseudoinclusion, ×100.

### 1.1 Cytologic Interpretation

Cytologic analysis is suggestive of metastasis to the lymph node, with the main differential diagnosis being thyroid carcinoma metastasis.

Evaluation of the right superficial cervical lymph node revealed high cellularity, predominantly composed of epithelial cells with moderate pleomorphism, arranged individually or in acinar and palisade patterns. Cytoplasmic borders were variably distinct, with a moderate amount of cytoplasm that was mildly to moderately basophilic. The nuclei were occasionally naked, round to oval, peripheral to eccentric, with reticular to coarse chromatin and variably prominent single to multiple nucleoli. There was moderate anisocytosis, anisokaryosis, occasional bi‐ and multinucleation, karyomegaly, typical mitotic figures (0–1/40× objective), and the occasional presence of intranuclear pseudoinclusions, which were round to oval and sharply defined within the nucleus. Additionally, a small heterogeneous population of lymphocytes was observed, predominantly small lymphocytes, followed by medium and large ones, containing cytoplasmic fragments in the background. The cytologic appearance was suggestive of metastasis to the lymph node, with the main differential diagnosis being thyroid carcinoma metastasis.

Cytologic analysis of the enlarged mandibular lymph node area revealed moderate cellularity, with a prevalence of epithelial cells exhibiting secretory features, arranged individually and in small clusters. Macrophages and eosinophilic amorphous material, suggestive of mucin, were observed. These cytologic findings, together with the history of a localized swelling, were suggestive of salivary gland hyperplasia (data not shown). In this case, inadvertent aspiration of normal salivary gland tissue could not be ruled out, as it was not possible to distinguish normal salivary tissue from hyperplastic tissue cytologically.

## 2 Additional Results

The patient had a history of mass removal from the right thyroid gland region 18 months prior to presentation. At that time, surgical resection of the mass was performed and sent for histopathologic examination, with a preliminary diagnosis of undifferentiated carcinoma. To determine the cell origin of the poorly differentiated neoplasm, immunohistochemistry (IHC) was performed using 3,3‐diaminobenzidine (DAB) and hematoxylin counterstaining. The neoplastic cells immunoreacted with: cytokeratin (AE1–AE3), thyroid transcription factor‐1 (TTF‐1), chromogranin, synaptophysin, and calcitonin. The proliferation marker Ki‐67 was positive in approximately 20% of the neoplastic cells. In conclusion, the immunohistochemical and morphologic profile confirmed the diagnosis of medullary thyroid carcinoma (parafollicular cell/C cell tumor).

Following the interpretation of the right superficial cervical lymph node and the enlarged mandibular lymph node area, the patient underwent surgical excision of these lesions. Subsequently, the samples were subjected to histopathologic analysis, where the diagnosis of metastatic medullary thyroid carcinoma (Figure 2A) and salivary gland without alterations (data not shown) was concluded. The tissues were sent for IHC, where the cells immunoreacted with TTF‐1 (Figure 2B), chromogranin, synaptophysin, AE1–AE3 (Figure 2C), and calcitonin (Figure 2D). The proliferation marker Ki‐67 was positive in approximately 20% of the neoplastic cells. The IHC findings for the neoplasm in the lymph node were the same as those for the original tumor previously excised, confirming metastatic medullary thyroid carcinoma.

**FIGURE 2:**
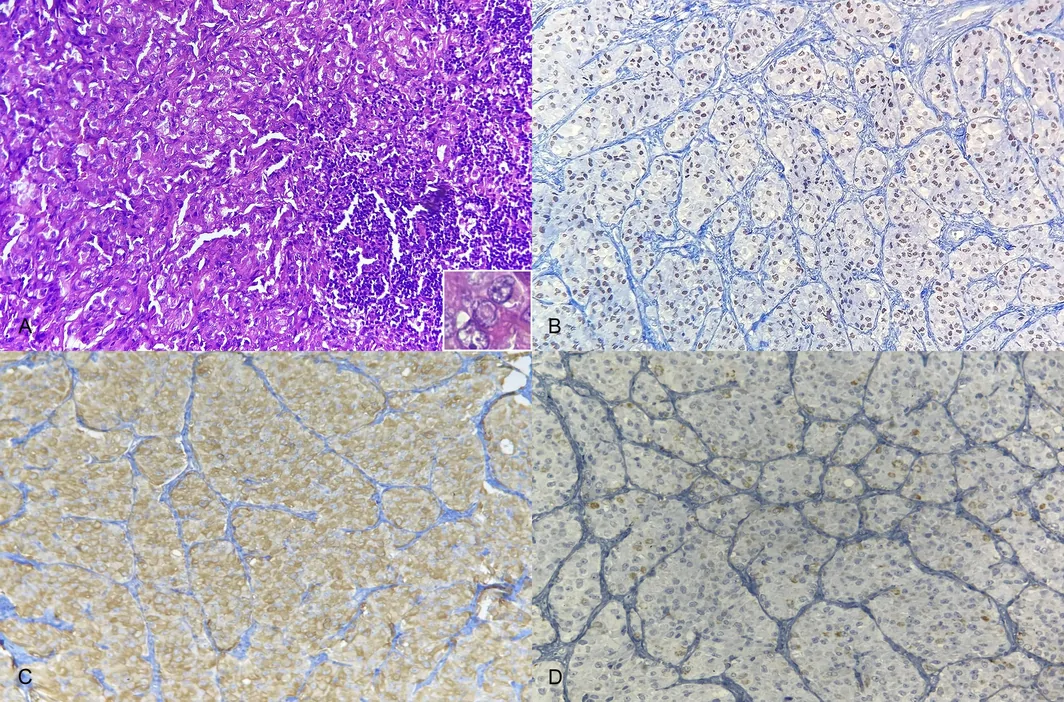
Metastatic medullary thyroid carcinoma from superficial cervical lymph node in an 8‐year‐old spayed female Maltese dog. (A) Histologic section. Neoplastic cells are large, round to polygonal, with a moderate to large amount of weakly eosinophilic and finely granular/reticulated cytoplasm, with poorly defined boundaries. Occasionally, intranuclear pseudoinclusions are observed. Pleomorphism and atypia are moderate. At the periphery of the neoplasm, there are clusters of lymphocytes (interpreted as remnants of nodal parenchyma). H&E stain, ×20 objective. Inset highlights an intranuclear pseudoinclusion, ×100. (B) The tumor cells reveal positivity for TTF‐1, ×20 objective. (C) The tumor cells exhibit granular cytoplasmatic positivity for calcitonin, ×20 objective. (D) The tumor cells show granular cytoplasmatic positivity for AE1–AE3, ×20 objective. Immunohistochemistry was performed using an immunoperoxidase method.

## 3 Discussion

Thyroid tumors represent approximately 1%–4% of all diagnosed neoplasms, being the most common endocrine malignancy in dogs, with no sexual predisposition in this species. Boxer, Golden Retriever, and Beagle breeds are overrepresented, and thyroid tumors occur more frequently in older dogs [1, 2, 3]. Follicular cell tumors are the most common, with the vast majority being malignant. However, carcinomas of medullary origin are less frequently reported, along with benign neoplasms such as adenomas [3, 4]. Local invasive growth often prevents complete surgical excision. In a study involving 144 dogs that underwent thyroidectomy, metastases were identified at admission in 12 dogs (8.3%). Among the 29 dogs that died due to thyroid cancer, 27.6% had metastases [3].

The most frequent clinical signs in dogs are associated with the involvement of structures adjacent to the thyroid gland, such as coughing, tachypnea, dyspnea, dysphonia, and dysphagia, in addition to the presence of a mass along the midline of the neck, which may be present from the base of the tongue to the base of the heart [5]. Most dogs with thyroid neoplasms do not present clinical signs related to abnormalities in the hormonal production of the gland [5].

A combination of complementary tests is required to diagnose thyroid neoplasms. On palpation of the region, cervical enlargement is often observed. Laboratory tests such as FNB, serum thyroid‐stimulating hormone (TSH) levels, thyroxine (T4), and triiodothyronine (T3) concentrations to identify underlying thyroid dysfunction, and histopathologic analysis for confirmation of malignancy are also used. However, in the present case, hormonal assays were not performed due to unavailability. Imaging studies such as ultrasound or computed tomography (CT) can detect multiple nodules not discernible by palpation and assess nodule vascularity, while scintigraphy may be used to detect functional or ectopic thyroid tissue and, in some cases, to evaluate disease extent [1, 5].

Immunohistochemical examinations are needed to determine the cell origin of the neoplasm (follicular vs. medullary) and to assist in the diagnosis of inconclusive or undifferentiated cases [4]. Histopathologic examination may be required to confirm malignancy when cytopathology is inconclusive, as cytologic features alone are often insufficient. This is particularly important because morphologic features of follicular and medullary carcinomas may overlap, making cytologic differentiation difficult or even impossible [4]. Distinguishing between these two tumor types holds clinical significance, as it may guide therapeutic strategies and reflect variations in tumor aggressiveness and prognosis [4].

In a comparative study of medullary thyroid carcinoma (MTC) and follicular thyroid carcinoma (FTC), calcitonin and thyroglobulin were the primary immunomarkers distinguishing the two, with calcitonin being positive in MTCs and thyroglobulin in FTCs [4]. In this case, cells exhibited immunoreactivity for calcitonin, confirming the MTC diagnosis. However, thyroglobulin testing was not possible due to the antibody's unavailability in the laboratory where the immunohistochemical tests were performed. Other immunomarkers, such as AE1–AE3, TTF‐1, and Ki‐67, showed no significant differences in determining the origin of the neoplasm [4].

Nuclear pseudoinclusions are cytoplasmic invaginations commonly observed in various types of tumors and are not exclusive to thyroid tumors. The presence of nuclear pseudoinclusions in canine thyroid carcinoma was reported by Bertazzolo et al. [6]. In contrast, true nuclear inclusions represent the accumulation of abnormal substances within the nucleus, such as viral particles or cytoplasmic components like immunoglobulins [7]. In the present case, the structures observed in hematoxylin and eosin (H&E) stained sections were designated as nuclear pseudoinclusions based on their sharply outlined, round to oval, eosinophilic appearance within the nucleus—a morphology consistent with descriptions found in human pathology literature [7]. Macronucleoli were not considered because the morphology observed was not compatible with nucleolar features. Although no ultrastructural or immunohistochemical studies were performed to confirm their nature, the term pseudoinclusion was chosen to reflect the current interpretation that these features most likely represent cytoplasmic invaginations rather than true nuclear inclusions. Their function and clinical significance remain unclear.

Overall, this case highlights the utility of cytology in diagnosing thyroid tumors and lymph node metastasis, as well as the importance of a thorough clinical history. It further illustrates how cytology can be a powerful tool for obtaining a likely diagnosis before confirmatory histopathologic analysis and immunohistochemical staining. The presence of nuclear pseudoinclusions is also noted as an interesting feature. After the surgical removal, the patient did not return for a follow‐up, limiting continued clinical monitoring.

## Funding

This work was supported by Conselho Nacional de Desenvolvimento Científico e Tecnológico and Coordenação de Aperfeiçoamento de Pessoal de Nível Superior.

## Conflicts of Interest

The authors declare no conflicts of interest.
